# Challenges in understanding and communicating the risk of zoonotic disease spillover from wild animal meat

**DOI:** 10.1093/pnasnexus/pgaf364

**Published:** 2025-11-13

**Authors:** Natalie Yoh, Gebbiena M Bron, Amy Ickowitz, Charlotte Spira, Lucy G Thorne, Pierre Nouvellet, Daniel J Ingram

**Affiliations:** Durrell Institute of Conservation and Ecology, School of Natural Sciences, University of Kent, Marlowe Building, Giles Lane, Canterbury CT2 7NZ, United Kingdom; Department of Animal Health, Animal Welfare, and Food Safety, Norwegian Veterinary Institute, P.O. Box 64, Ås 1431, Norway; Center for International Forestry Research and World Agroforestry, CIFOR Headquarters, Situ Gede, Bogor Barat, Bogor 16115, Indonesia; Center for International Forestry Research and World Agroforestry, CIFOR Headquarters, Situ Gede, Bogor Barat, Bogor 16115, Indonesia; Department of Infectious Disease, Imperial College London, Norfolk Place, London W2 1PG, United Kingdom; Department of Ecology and Evolution, School of Life Sciences, University of Sussex, Falmer, Brighton and Hove BN1 9RH, United Kingdom; MRC Centre for Global Infectious Disease Analysis and Abdul Latif Jameel Institute for Disease and Emergency Analytics, Imperial College London, White City Campus, 90 Wood Lane, London W12 OBZ, United Kingdom; Durrell Institute of Conservation and Ecology, School of Natural Sciences, University of Kent, Marlowe Building, Giles Lane, Canterbury CT2 7NZ, United Kingdom

**Keywords:** One Health, emerging infectious disease, bushmeat, game, food safety

## Abstract

Discussions around managing hunting and the consumption of wild animal meat increasingly emphasizes public health concerns and the risk of zoonotic spillover. In this article, we explore factors that may lead to under- or overestimating health risks from wild meat and break down key terminology for a multidisciplinary audience. We outline key principles of disease ecology and epidemiology that are often overlooked when quantifying spillover risk, and reflect on the importance of contextualizing health risks relative to food-health systems more broadly. We discuss how misrepresenting risks, intentionally or unintentionally, to justify conservation practices can have unintended negative conservation and public health consequences—despite the importance of conservation in protecting human health more broadly. We stress the importance of considering individual and local health outcomes (food security, neglected tropical diseases, etc.), not only those impacting global health (i.e. pandemic prevention). Finally, we advocate for evidence-informed, context-appropriate strategies for wild meat management.

## Introduction

From research and policy to social media, there are growing concerns about the potential health risks from **wild meat** (i.e. the meat and other body parts of nondomestic, free-ranging animals) (see Table 1). Concerns often focus on the risk of **spillover events** associated with zoonotic **emerging infectious diseases**, the majority originating from wildlife (9). An estimated 60% of all human **pathogens** have been linked to zoonotic spillover events from domestic or nondomestic species (10, 11). Zoonotic spillover events can represent isolated or periodic events and can cause a range of health outcomes: from benign **infections** to **diseases** with high individual morbidity/mortality but limited onward transmission, to large-scale **communicable** disease **outbreaks**, including COVID-19 and Ebola virus disease. Caused by SARS-CoV-2, COVID-19 has resulted in an estimated >27 million deaths worldwide (12). Meanwhile, Ebola virus disease is notorious for its high case fatality rates between 30 and 90% across past outbreaks (13). Both diseases illustrate how a pathogen spillover event can lead to snowballing human-to-human transmission, resulting in regional or global outbreaks, and have both been suppositionally linked with wild meat. Such outbreaks can strain public healthcare systems, cause widespread illness and loss of life, destabilize governance and public trust, and can cause economic recessions following movement and trade restrictions (14, 15). The consequences associated with a spillover event vary widely across this continuum. Accurately understanding and transparently describing the risks associated with spillover events is key for designing effective mitigation strategies to reduce the human burden of disease.

**Table 1.** Glossary of terms.

Term	Definition
**Consumption and contamination**	**Consumption:** Ingestion of food, water, or other material. **Contamination**: Indirect contact transmission, in which the hazard has been transferred from a source to a previously nonhazardous object.
**Dead-end hosts and dead-end events**	**Dead-end host:** A host that can be infected with an infectious agent but cannot or has limited capacity to transmit the agent to new hosts. **Dead-end events:** Spillover events into dead-end hosts.
**Disease, infectious disease, and communicable disease**	**Disease:** A set of clinical symptoms and signs of illness resulting from damage and disruption to host tissues. The cause of disease can be from infectious agents. **Infectious disease:** A disease caused by an infection from a pathogen. The pathogen may or may not be transmissible from person to person. **Communicable disease:** An infectious disease that can be transmitted between people.
**Emerging and re-emerging infectious diseases**	Diseases that have recently emerged in a population. This can represent diseases already known but that are rapidly increasing in incidence or geographic range, or diseases that have recently been discovered for the first time.
**Epidemiology**	The study of the distribution and determinants of health including disease, and the application of this study to the control of diseases and other health problems.
**Epizootic**	An outbreak of a disease within an animal population.
**Exposure**	The point at which a potential host comes into contact with an infectious agent. Exposure may or may not lead to an infection.
**Foodborne diseases**	Diseases caused by consumption or exposure to contaminated food and drink at any stage of the food supply chain. Foodborne diseases may or may not be caused by pathogens capable of zoonotic transmission.
**Hazardous**	A substance, activity, or condition that poses a risk to a person's health or safety. Foodborne hazards include biological hazards (i.e. pathogens), chemical hazards (e.g. mycotoxins, heavy metals), physical hazards (e.g. sharp objects), or allergens.
**Host species**	A species that can harbor an infectious agent, either internally or externally. The agent may not necessarily be able to replicate in the host and may or may not cause disease. The host may not be capable of maintaining the pathogen in nature, be essential in an agent's life cycle, or be able to transmit the agent to new hosts. In contrast, see Reservoir/maintenance hosts.
**Host specificity and susceptibility**	**Specificity:** The diversity of host species that an agent is capable of infecting. **Susceptibility:** The ability of an individual to resist infection or limit disease given exposure.
**Incidence rate**	The number of new cases in a population over a given period of time.
**Infection**	An agent has established within the host tissues. Infection does not always result in disease (i.e. clinical manifestation).
**Infectious agent and pathogen**	**Infectious agent:** A microorganism or other biological agent that can cause an infection in a host organism. These include viruses, bacteria, fungi, protozoa, parasites, and prions. **Pathogen:** An infectious agent that can cause disease or illness in a host organism.
**Infectivity (of a pathogen)**	The likelihood that an agent will infect a host given exposure.
**Neglected tropical disease**	Diseases that disproportionately affect people living in impoverished communities and that are mainly prevalent in tropical areas. As such, they often receive disproportionately less research attention and investment than other human diseases. They include diseases caused by viruses, bacteria, parasites, fungi, and toxins, and include both zoonotic diseases and foodborne diseases. They are responsible for devastating health, social, and economic consequences in many tropical countries.
**Outbreak, epidemic, and pandemic**	These 3 terms refer to the geographic scope of the disease, not the disease severity: **Outbreak:** An increase in cases of a particular disease or other specific health-related behavior in a population at a local or regional scale above expected occurrence rates. **Epidemic:** An outbreak of disease that impacts humans over larger spatial scales, affecting multiple regions or countries. **Pandemic:** A global outbreak of a disease.
**Pathogenicity**	The ability of an agent to cause disease given infection.
**Prevalence**	The proportion of a population infected by a particular agent at a specific point in time or a given time period.
**Reservoir (of infection), reservoir/maintenance host, and environmental reservoir**	**Reservoir:** Definitions regarding what constitutes a reservoir remain inconsistent. In this paper, we define a reservoir as one or more epidemiologically connected populations or environments in which an agent can be permanently maintained and from which infection is transmitted to another susceptible host species. This secondary host may or may not develop disease. **Reservoir host:** A host species that maintains an agent in nature, often with no effect on their fitness. Agents may be affiliated with a single host species (e.g. a primary reservoir species) or several host species may act as reservoirs. **Environmental reservoir:** Nonanimal reservoirs, typically nonliving habitats, which maintain agents outside of hosts and vectors. Environmental reservoirs can transmit agents to new hosts. Diseases caused by zoonotic agents that are maintained by environmental reservoirs are known as saprozoonoses.
**Reverse spillover and Spillback**	**Reverse spillover:** The transmission of an agent from humans to animals (including wildlife) in which humans can be a reservoir species. **Spillback:** The cross-species transmission of an agent from a host species back to a previously infected host species. This term often relates to reverse zoonoses in which the agent originally had a zoonotic origin.
**Surveillance: general surveillance, targeted surveillance, and untargeted surveillance**	**General surveillance:** A top-down approach to monitoring health threats, starting with signs of illness in a population, identifying the causal agent, and then identifying the source/method of exposure. **Targeted surveillance:** Monitoring specific populations or environments to detect known zoonotic agents, infectious agents, and health threats. This approach often involves systematic sampling of seemingly healthy populations to understand host-agent relationships, determine agent prevalence, and assess the likelihood of disease emergence. **Untargeted surveillance:** Broad, nonspecific monitoring that does not focus on predefined zoonotic health threats or specific host species. This approach is well suited to identifying novel infectious agents or host-agent relationships.
**Spillover events**	An event during which an agent from one species infects another species. A zoonotic spillover refers specifically to animal-to-human transmission.
**Vector and vector-borne diseases**	**Vectors:** Definitions regarding what constitutes a vector remain inconsistent. In this paper, we define vectors as invertebrates that act as carriers to transport infectious agents between vertebrate hosts through biological or mechanical transmission. However, the definition of a vector can vary greatly across studies with the broadest definition encompassing any organism (vertebrate or invertebrate) that can act as a carrier of an infectious agent between other organisms. **Vector-borne diseases:** Diseases caused by an infectious agent that can be transmitted between hosts via (invertebrate) vectors.
**Virulence**	A measure of disease severity given infection (i.e. a decrease in host fitness associated with an infection).
**Vulnerability:** **clinical/medical/structural/socioeconomic**	**Clinical vulnerability:** an individual's risk of negative health outcomes based on internal factors such as age, gender, ethnicity, pre-existing medical conditions, and pregnancy. **Structural vulnerability:** An individual's risk of negative health outcomes based on external structures (i.e. socioeconomic, political, and cultural conditions) that affect their ability to access healthcare and pursue a healthy lifestyle.
**Wet market**	Markets in an open-air or partially open-air setting often comprising individual vendor stalls offering consumption-oriented, perishable goods (i.e. fresh meats and produce). Markets range from those exclusively selling fruits and vegetables to those selling fresh or preserved meat, or live animals for consumption. Meat or live animals can represent domestic, captive-bred, or wild-caught animals. *Wet market* typically refers to markets in an Asian context. Wet markets do not necessarily sell wild animals or their meat.
**Wild meat, bushmeat, and game**	**Wild meat:** The meat and other body parts of wild terrestrial and aquatic animals (excluding fish) used for food. **Bushmeat:** The meat and other body parts of wild terrestrial vertebrates used for food, typically in sub-Saharan Africa. **Game:** The meat and other body parts of wild terrestrial vertebrates used for food or sport hunting, typically in Europe, North America, and Australasia.
**Wildlife disease**	Animal diseases that affect free-roaming/non-domesticated species. These diseases may or may not be caused by infectious agents that can infect humans.
**Zoonotic origin**	Infectious agents that originated from animal hosts.

Please note the definitions here represent a common consensus across multiple sources but may differ from other sources. **Sources:** van Seventer and Hochberg 2017 (1); Haydon et al., 2002 (2); Salkeld et al., 2023 (3); Quesada et al., 2011 (4); Wilson et al., 2017 (5); World Health Organization 2024 (6); Lin et al. 2021 (7); Ingram et al. 2021 (8).

In response to COVID-19 and Ebola virus disease outbreaks, many governments increased enforcement of illegal wildlife trade. Several also instigated temporary or permanent bans on otherwise legal trade and consumption at local, regional, or national scales (16–18). These bans range from blanket bans on all wildlife markets to species-specific bans, e.g. prohibiting the sale of bats and pangolins, for taxa purported to be high risk (19). In some cases, conservation organizations have leveraged these public health concerns to garner support for restricting or banning hunting. However, wild meat can be important for food security and health in some communities (20, 21) and plays an important cultural component in many people's lives (22). For others, hunting is a recreational activity and wild meat is a food preference, not a necessity. Motivations for, and practices of, consuming wild meat also differ greatly across communities. For example, between consumption for subsistence by Indigenous, tribal, or traditional peoples, urban or rural consumers, and consumption related to local, regional, or global market chains. These contexts may include differences in which species are consumed.

Risk mapping to identify possible “hotspots” for zoonotic spillover from wild meat is useful in guiding policy, funding, and management action (23, 24). However, accurately assessing risk relies on cross-disciplinary understanding of **epidemiology**, landscape ecology, food systems, and social behavior, among others. Language remains a major barrier to collaborations across disciplines where terminology may be inconsistent, highly technical, or poorly defined. For example, wild meat, **game**, and **bushmeat** are all used to describe meat from wild animals in different geographical regions. Differences in terminology can lead to biases when mapping risk and prejudice perceptions toward food practices in particular regions. Whether a pathogen is considered zoonotic can also differ depending on the definition used (25). Even more simply, whether research refers to **infectious agents** versus pathogens, pathogens versus diseases, or **host species** versus a **reservoir species** can be confusing for researchers from nonmedical or nonveterinary backgrounds and misinterpreted. For example, Woolhouse and Gowtage-Sequeria (10), Jones et al (9), and Taylor et al (11) are frequently misquoted as stating that approximately 60% of human diseases (i.e. all diseases that cause illness in humans, including those without infectious origins) have a zoonotic origin or 75% of emerging infectious diseases (that cause illness in humans) are zoonotic. Rather, these studies state that 58% to 61% of human pathogens have a zoonotic origin (10, 11), 60% of emerging infectious disease spillover events are caused by zoonotic pathogens (72% of which originate from wildlife) (9), and 75% of emerging pathogens have a zoonotic origin (11).

Effective communication also depends on a mutual understanding of what constitutes risk, a factor often overlooked. Is risk defined as the diversity of infectious agents associated with animals in a food supply chain; the likelihood of a spillover occurring; or the potential health risks associated with a spillover event, for individual, local, and global human health? Different measures provide different information for decision making. From this point forward, we define risk as the likelihood of exposure to a hazard (i.e. a potential source of harm; an infectious agent) and the likelihood and impact of harm to human health given exposure (e.g. the resulting infection or illness).

This study addresses 3 critical challenges in accurately communicating and assessing health risks from zoonotic spillover connected to wild meat: (1) scientific and methodological challenges in defining and quantifying zoonotic disease risk from wild meat, (2) contextual factors that influence actual risk exposure and health outcomes in food systems, and (3) implications for risk management and the need for evidence-based management strategies. We break down key terminology and principles for a multidisciplinary audience and demonstrate how differences in how we define key terms, quantify risk, and contextualize the results within larger food-health systems frame our perceptions. This may ultimately lead to under- or overestimating health risks, intentionally or unintentionally, at local or global scales. We explore differences between pathogens that predominantly impact individuals (e.g. foodborne bacteria or parasites) versus pathogens with greater relevance for “global” health (e.g. respiratory RNA viruses) and echo concerns that misrepresenting risk will lead to negative conservation and health outcomes, and erode public trust in national and international institutions (24, 26). Finally, we provide guidance for future research to ensure transparency and avoid miscommunication or fear mongering.

### Scientific and methodological challenges

#### Defining what is meant by zoonotic

Quantifying risks to human health requires defining what we mean by *zoonotic* or *zoonosis*/*zoonoses*. Broadly, a zoonosis may be characterized as a pathogen, infection, or disease that originated in animal populations (i.e. zoonotic origin) and is known to—or has the potential to be—transmitted from nonhuman animals to humans (i.e. zoonotic transmission). However, there is a great deal of discrepancy in how these terms are used. As highlighted by Singh et al. (25), the World Health Organization lists 4 definitions of a zoonosis/zoonoses; differing in terms of the relevant species included (nonhuman animals versus exclusively vertebrates), the direction of transmission (i.e. unidirectional from nonhuman animals to humans or bidirectional between nonhuman animals and humans), whether transmission occurs naturally, and whether it pertains to an infection (or agent thereof) versus a disease. Definitions may also differ depending on the evidence used to determine whether an agent/pathogen is capable of animal-to-human transmission and/or causing disease, whether (invertebrate) **vector-borne** pathogens or those with **environmental reservoirs** should be included (e.g. Zika virus, anthrax), and whether a pathogen requires ongoing spillover events to maintain human infections (Box 1). However, inconsistencies are not necessarily apparent as many studies do not state how they define zoonoses.

Box 1. Example of a disease with both zoonotic and non-zoonotic transmission. There were an estimated 249 million malaria cases globally in 2022, resulting in approximately 608,000 fatalities (27). The disease is caused by protozoan parasites of the genus *Plasmodium*. From over 200 *Plasmodium* spp., five are known to frequently infect and cause malaria in humans, *Plasmodium falciparum*, *P. vivax*, *P. malariae*, *P. ovale*, and *P. knowlesi*. They are transmitted exclusively by *Anopheline* mosquitoes. Four species do not require nonhuman vertebrate hosts to complete their lifecycle and are transmitted primarily between human hosts (so-called non-zoonotic malaria). In contrast, *P. knowlesi* is transmitted mainly between Southeast Asian primates but can cause so-called zoonotic malaria in humans (28). Other simian *Plasmodium* species, e.g. *P. cynomolgi*, *P. simium,* and *P. brasilianum*, have also resulted in rare natural cases of zoonotic malaria (29–31). Cases of zoonotic malaria are rising and now represent the sole cause of malaria in regions previously declared malaria-free. However, the delineation between zoonotic and non-zoonotic malaria species is not always clear-cut. *P. vivax is* the second most prevalent *Plasmodium* sp. in humans. There are two distinct phylogenetic clades of *P. vivax* known to circulate in Africa: a “human clade” and a distinct clade circulating in great apes. Prugnolle et al. (32) showed that both clades can cause infection in apes and humans and have suggested that apes may serve as reservoirs for *P. vivax*. Current malaria elimination efforts focus on eradicating non-zoonotic malaria and thus do not address the risk of future re-establishment following zoonotic spillover events (33). Malaria is a good example of how we do not yet understand the dynamics underpinning some of the world's most important human infectious diseases.

#### Risk assessment methods and limitations

**Fig. 1.**
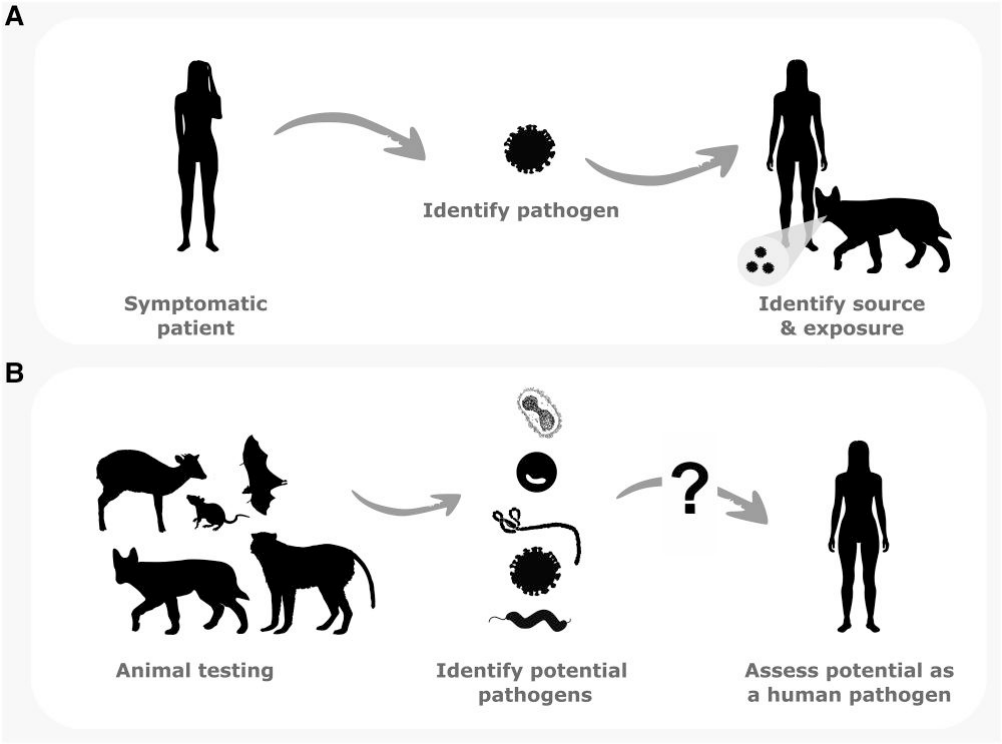
Simplified approaches for identifying zoonotic pathogens and their reservoir hosts. (A) General surveillance of human illness and (B) untargeted or targeted surveillance of wildlife populations.

There are three main approaches for identifying and quantifying the number of infectious agents associated with a given host species, and the corresponding risk those agents may have on human health (Fig. 1). Researchers can work backward from a known symptomatic infection in humans and identify the pathogen and its relevant hosts (34). This approach provides strong evidence that an agent has clinical relevance to human health. However, this **general surveillance** of known infections may underestimate the number of zoonotic pathogens present in a population given many infections cause minor symptoms or are not documented, especially in regions with limited access to healthcare. It can also be very difficult to postcircumstantially identify which animals were involved in a spillover event, as demonstrated by SARS-CoV-2 and Ebola virus (3, 35). Alternatively, researchers can use **untargeted** or **targeted surveillance** to sample seemingly healthy animals, identify potential agents of infection, and predict their likelihood of zoonotic transmission (36). These two surveillance approaches can provide a greater understanding of the distribution and evolutionary relatedness of microbial agents across species. Where possible, researchers may assess factors such as the capacity of novel agents to replicate in human cells, the likelihood of the agent causing disease, and the likelihood of human-to-human transmission. However, these approaches likely overestimate the number of agents of clinical relevance to humans as many viruses, bacteria, parasites, etc. result in the same disease, cause minimal illness, or are **non-pathogenic**. Moreover, these approaches do not necessarily consider the mode of transmission and other factors that prevent **exposure** to a pathogen. Ultimately, all three approaches have strengths and weaknesses that are important to caveat for researchers to avoid miscommunicating risk.

The information needed to understand the zoonotic potential of most infectious agents is incomplete. In particular, we often lack knowledge of the **pathogenesis** and epidemiology of many **wildlife diseases** (37), i.e. the determinants underpinning the distribution and prevalence of agents within animal populations and the processes by which an infection causes disease. To mitigate these knowledge gaps, infectious agents are grouped based on phylogeny, with the assumption that closely related agents share similar zoonotic transmission risks and health outcomes (38, 39). Grouping agents can be useful for predicting the pool of potential hosts for host-generalist pathogens capable of infecting and causing disease in a wide range of animal species, such as rabies lyssavirus. However, many pathogens have a narrow host range, and although they may cause disease in one species, they may not in another (40). Several filoviruses are highly pathogenic in humans (e.g. including Ebola virus, Sudan virus, and Marburg virus); however, others are not known to cause any adverse health effects in humans (e.g. Reston virus and Bombali virus) (41). On a molecular level, **host specificity** is driven by a range of interactions between pathogen and host biology. In the case of viruses, these determinants include how and where a virus can bind and enter a cell, as well as whether the virus can replicate using the new host cell machinery and overcome the host's immune system, all of which can impact the resulting symptoms. When quantifying the number of novel infections or diseases, it is important to consider that multiple pathogens may cause the same clinical presentation or disease (10). Therefore, identifying novel agents does not necessarily equate to more diseases but may impact treatment and prevention strategies (Box 1). In many cases, pathogens require a chain of minor and major evolutionary adaptations to be able to infect new hosts (including humans) and sufficient opportunity for this evolution to occur [e.g. Bansal et al. (42)]. The factors that predict a **host's susceptibility** and the relationship between pathogenesis/**virulence** and transmission routes are actively researched. However, there is not one universal way in which evolutionary relatedness impacts pathology, and an oversimplified grouping of agents (or hosts) can lead to misrepresentation of spillover risk and potential health outcomes.

### Contextual factors influencing health outcomes

#### Exposure through meat supply chains

The supply and consumption of wild meat provide multiple opportunities for pathogen spillover (43, 44). Wild meat consumption is a global phenomenon; however, consumption in much of Europe and North America remains infrequent or largely associated with hunters and their social connections, or local and high-end specialized restaurants. In contrast, wild meat consumption in subtropical and tropical regions, and among Indigenous/traditional peoples, is more commonplace, although motivations differ greatly across regions and products, as does the role of market chains (8). Hunting, handling, butchering, transport and storage, food preparation, market exchange, and consumption all provide pathways for potential spillover events, including for emerging or reemerging infectious diseases. Since 1996, the number of journal articles investigating zoonotic disease risks linked to wild meat has risen, with 37% published since 2020 (45). However, given the complexities of infectious disease and food systems, our understanding of risk remains limited.

Several recent studies have compiled available data to summarize the current understanding of the links between wild meat and known pathogens (46–50), as well as zoonotic disease risk more broadly (9, 51, 52). Such studies use lists of agents/pathogens affiliated with host species of interest to assess geographic and taxonomic disease risk based on phylogeny, diversity, and distribution. It is rarely possible for these studies to consider infection **prevalence** and therefore the likelihood of encountering an infected individual is treated as equal across species, pathogens, and time. Studies often focus on tropical regions explicitly, given that these are perceived as higher risk (49, 50), or by using search terms that disproportionately correspond to certain geographic regions [e.g. bushmeat; as acknowledged in Moloney et al. (48)]. Despite this, bacterial spillover events have most frequently been documented in North America, and twice as many spillover events have been documented in Europe compared with South America (47). In Europe, most studies focus on endemic **foodborne diseases** (such as salmonellosis), whereas research in Africa largely focuses on emerging/reemerging viral infectious diseases (e.g. Ebola virus disease) (45). These geographic differences partly reflect not only differences in disease surveillance and reporting capacity, but also motivations behind surveillance and study efforts.

#### Wild versus domestic animals

**Fig. 2.**
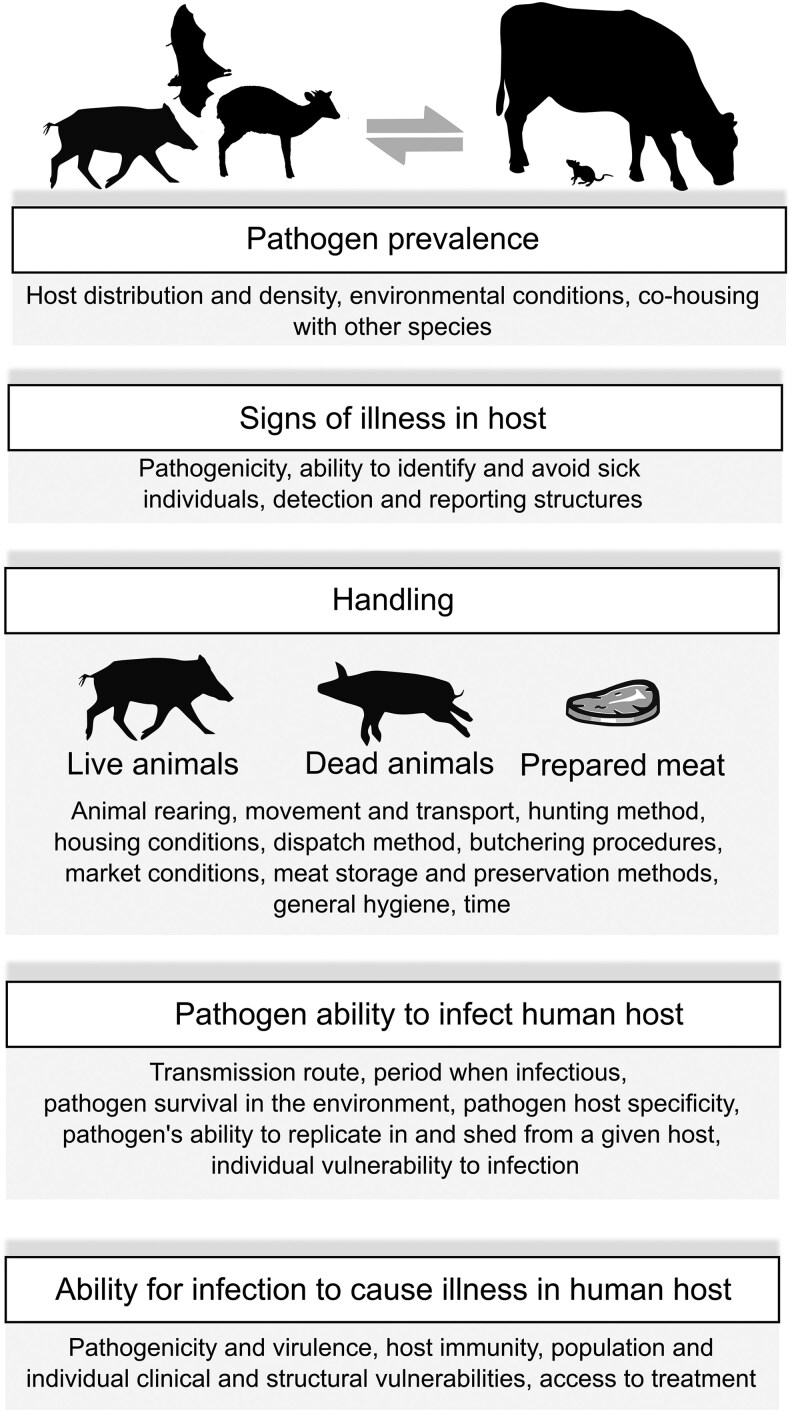
Examples of factors to consider when assessing the risk of human exposure to animal pathogens, and the subsequent risk to an individual's health, in meat supply chains. Animals in the meat supply chain may vary from non-domesticated species to semi-domesticated to domesticated. Animals may also represent captive-bred, captive wild-caught, or captive-reared.

**Fig. 3.**
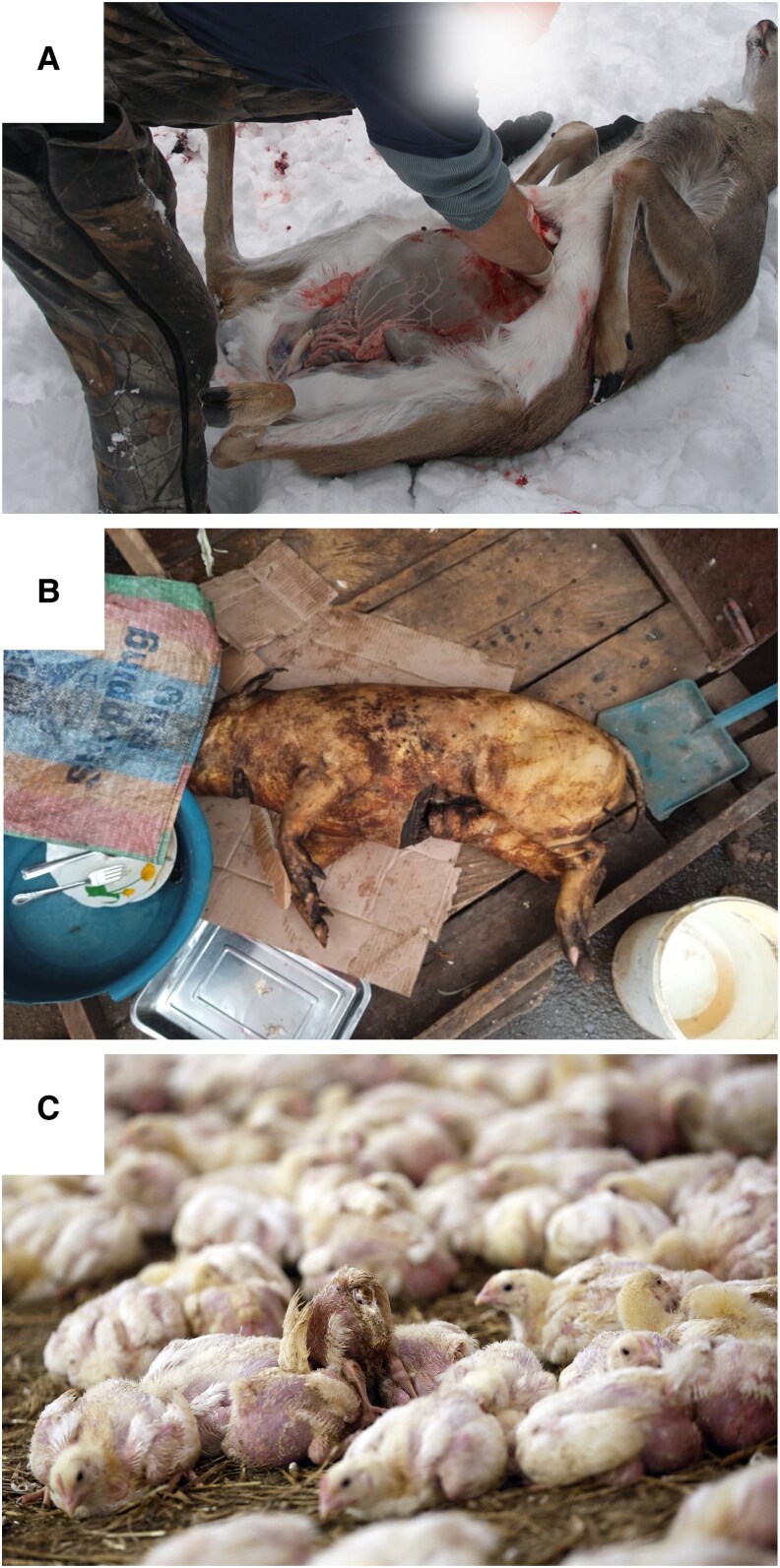
Three examples of food safety contexts across the continuum of meat supply chains, from no market chain to wholesale production. All three contexts require an understanding of food hygiene and disease transmission to implement control measures necessary to avoid the possibility of spillover. (A) Field dressing of a locally hunted white-tailed deer (*Odocoileus virginianus*) in Michigan, USA [Photo by MJCdetroit, CC-BY-SA-3.0, via Wikimedia Commons, edit: face obscured]. (B) A roasted pig for sale left uncovered at a market in Yaoundé, Cameroon, either a domestic or wild-sourced pig [Photo by Gertruide Dalila Massoh]. (C) Industrial-scale, commercial broiler chicken (*Gallus gallus domesticus*) rearing facility in Europe illustrating high-density, mass production [Photo by Otwarte Klatki, CC-BY-2.0, via Wikimedia Commons].

Wildlife is not the only source of zoonotic pathogens (Fig. 2). Domestic animals can constitute important reservoirs and intermediary hosts, particularly those for food production (53). Animal husbandry plays an important role in determining the likelihood of zoonotic spillover from domestic animals by affecting infection prevalence within a population. For example, overcrowding has a two-fold impact on disease spread; stress compromises an animal's immune system, increasing the likelihood of infection, and cramped conditions increase the likelihood of exposure (54) (Fig. 3). Hence, intensive agricultural practices can cultivate large-scale **epizootic** outbreaks and opportunities for a pathogen to evolve, and introduce new exposure pathways for spillover into human populations and wildlife. Recent human cases of H5N1 avian influenza A have been associated with widespread outbreaks of the virus in domestic cattle across dairy farms in the United States, sparking concerns of a newly emerging public health threat (55). The handling and transport of wildlife, and the configuration of wild meat markets, can also determine the likelihood of intra- and interspecific species disease spread, directly or via bodily fluids between living and dead, or domestic and wild animals (7, 56). Pathogen prevalence is only one factor impacting the risk of spillover. Risk also depends on factors including exposure (i.e. the type, length, and frequency of exposure), the environmental stability and **infectivity** of a pathogen, and any barriers to infection. The likelihood of exposure and the resulting probability of infection differ across sociodemographic groups and their respective roles in meat consumption and supply chains. In the United States, farm workers currently represent most cases of H5N1 infection (55). Farm workers in the United States, often migrant or seasonal workers, face health inequities related to unsafe working conditions, crowded or unsafe housing, their immigration status, and limited access to healthcare, among other factors (57, 58). Such conditions create an ideal environment for viral spillover and adaptation with a low risk of detection.

#### Food safety and exposure factors

Most consumers do not come into contact with live animals, rather **contamination** and **consumption** are the main exposure pathways. All meat is potentially **hazardous** when prepared and stored incorrectly. Foodborne disease causes approximately 600 million cases of illness and 420,000 documented deaths worldwide annually (59), predominantly affecting individual health or health at a local scale. Lower-income countries disproportionately suffer the health burdens of foodborne diseases due to less access to safe foods, clean drinking water, and treatment (59). The risk of foodborne illness is affected by factors such as whether meat is fresh or preserved, access to clean water, sanitation, cold storage, and opportunities for cross-contamination (Fig. 3b). National food safety regulations aim to reduce exposure in the food supply chain by outlining minimum safety requirements for businesses and recommendations for consumers. Food safety practices vary worldwide but disease surveillance and traceability systems are much more established for formal commercial supply chains, typically dominated by livestock meat (56). Advocates for wild meat consumption argue that the risks of zoonotic transmission can be mitigated by enforcing similar meat hygiene practices used to prevent foodborne illnesses in domestic meat production (56). By the same rationale, Karesh et al. (60) argued that increasing livestock production to substitute wild meat may ultimately lead to higher pathogen emergence in countries that cannot apply appropriate disease-management practices. Regardless, we note that different species are not inherently interchangeable within food cultures (43, 61), and research comparing the relative safety of wild meat to domestic meat is limited outside of the Global North. It is unclear whether it is the species consumed (hazard) or the animal or food handling practices implemented (exposure) that have a greater impact on overall health risks. The Global North is not immune to the challenges of managing disease risk in meat supply chains (Fig. 3C). For example, inadequate hygiene and biosecurity measures have failed to prevent outbreaks of highly pathogenic avian influenza (HPAI) subtype H5N1 in US dairy cattle following farm-to-farm transmission (62). Continued transmission of HPAI H5N1 across US dairy herds represents a global human health concern given the pandemic potential of HPAI viruses (63).

#### Clinical and structural vulnerabilities

Studies that quantify risk based on the number of novel agents across species fail to consider their relevance for human health. Many known zoonotic infections present asymptomatically or cause mild illness in all or the majority of cases (41, 64). Nevertheless, a disease's severity, incidence, prevalence, and likelihood of human-to-human transmission need to be considered when quantifying risk to an individual or for local, regional, or global populations. For example, although *Toxoplasma gondii* predominantly causes no or mild symptoms in healthy individuals (65), due to the scale of infection, toxoplasmosis is one of the most clinically important parasitic zoonoses globally (66). A person's susceptibility to a given disease following exposure is shaped by a complex interaction of individual and environmental factors, including a person's **clinical** or **structural vulnerability**.

Malnutrition (undernutrition or obesity) can weaken the body's ability to combat infections, elevating an individual's risk of falling ill, and increasing the severity of symptoms (67). Infectious diseases such as tuberculosis can in turn worsen malnutrition (68). In this way, food insecurity and limited medical care can cause a cyclical pattern of illness in vulnerable communities which may otherwise be preventable or treatable. Infections may also go unreported in areas where health care is limited or in communities using alternative or traditional medicine options. Thus, it is important to consider potential vulnerabilities when predicting health outcomes for a given population and informing preventative strategies. Wild meat consumption contributes substantially to the food security and nutrition of people around the world but particularly in rural areas of the tropics. Wild meat provides important nutrients (e.g. iron, zinc, vitamin B12, and protein) that may be limited in people's diets (20, 69, 70), directly contributing to positive health outcomes. Restricting people's access to wild meat in these cases could exacerbate malnutrition, resulting in greater vulnerability to disease.

#### Perception of risk in food systems

Perceptions of risk not only impact people's willingness to consume wild meat but can also predict whether individuals engage in activities, such as butchering, and whether they follow food safety practices during preparation and consumption. Food safety concerns can act as a barrier to wild meat consumption, entirely or for certain species or animal parts, or circumstantially (71). In Poland, differences in risk perception impact willingness to consume wild meat outside of the home (72). However, for some consumers, meat from hunted animals is perceived as healthier than farmed meat (both from non-domesticated and domesticated species), as it is considered more natural, nutritious, and fresher, which can outweigh concerns surrounding exposure to pathogens (73–76). There may also be other factors influencing how someone engages with wild meat. For instance, many believe Nipah or Ebola virus disease are not natural phenomena, but rather spiritual ones (i.e. resulting from witchcraft or as punishment from God) (77, 78). Therefore, an individual may see no value in adhering to food safety practices. A survey of 2725 hunters in Colorado, USA, found that 42% believed that there was no risk to their health from chronic wasting disease given the lack of evidence to indicate this disease (which primarily affects ungulates) can also cause human illness—impacting adherence to safety legislation (Fig. 3A) (79). However, recognizing risk does not necessarily mean that people can or will choose to avoid it. In rural communities along the Kenya-Tanzania border, Patel et al. (80) found that 156 of 299 respondents were worried about diseases from wild meat, but only 21% had reduced their consumption. This lower-than-expected decrease was mostly due to costs and availability. “Risky” food may still pose a safer alternative to no food.

Public perception links the SARS-CoV-2 pandemic to a spillover event at the Huanan Seafood Wholesale market in Wuhan, China, in December 2019 (81). This narrative is circulated widely in public discourse and repeatedly written as fact in scientific literature. However, a joint World Health Organization (WHO)–China study determined that SARS-CoV-2 likely emerged earlier than reported but could not conclude when, where, or how it occurred (35, 82). To date (June 2025), the WHO has not found sufficient evidence to indicate that the outbreak originated in any market in Wuhan. Despite this, the blame was quickly attributed to wet markets (any market that sells fresh produce, that may or may not sell wild meat, typically associated with Asia) with media and politicians calling for their permanent ban (83–85). New studies continue to shed light on the origins and spread of SARS-CoV-2 (86, 87), but currently it is unknown whether the market was the source of the outbreak or acted as an amplifier for transmission given the high visitor turnover and crowded conditions. Scientific research is inextricably linked to the sociopolitical climate in which it is funded and undertaken. As such, previous work has highlighted the impact that racism and colonialism can have in food health and conservation research (88, 89). Hence, pre-existing bias and complexity can foster misrepresentation or misinterpretation of the “facts” regarding zoonotic spillover risk when uncertainty is not clearly explained (90, 91).

#### Conservation agendas in health messaging

Hunting for wild meat consumption is a leading cause of species’ population declines, particularly for mammals in tropical regions (92). Therefore, campaigns advocating banning wild meat consumption have been presented as “killing two birds with one stone”—benefiting species conservation and public health. Done well, education campaigns can become a useful part of preventative healthcare, empowering individuals with the knowledge to make their own healthcare decisions (though not necessarily leading to behavioral change) (93, 94). However, inappropriate health messaging advocating against wild meat consumption can clash with consumers’ lived experiences (95, 96). This disconnect can foster distrust between the public and institutions that may be perceived as using health messaging to prioritize wildlife protection over people's access to food, income, and culture. Inappropriate health messaging can have lasting legacies on public trust and damage the success of future health education campaigns beyond those related to conservation (97).

The conversation around the risks of eating wild meat rarely focuses on an individual hunter, vendor, or consumer. Many zoonotic diseases endemic in the Global South represent **neglected tropical diseases**, receiving little international attention as they are not seen as health threats to the Global North (61). Wild meat bans, however, are “marketed” to prevent future pandemics, potentially at the expense of food security (98). However, transmission connected to the wild meat supply chain is more likely to represent **dead-end events**, predominantly impacting the health of the individual. While there are many reasons a pathogen may not transmit human to human (e.g. insufficient replication), many pathogens simply lack suitable pathways for subsequent transmission (i.e. pathogens spread through animal bites or contaminated food). This does not negate the need for urgent and effective management and policy strategies to curb the risk of emerging infectious diseases (e.g. WHO Pandemic Agreement) (6). Campaigns focused on safe food handling and providing hygiene infrastructure (where appropriate) could help reduce the risk of common infections and foodborne illness, while also reducing the risk of spillover of newly emerging pathogens (23, 56, 99, 100). Improved food hygiene could also benefit conservation directly by reducing food spoilage (e.g. refrigeration helps traders store meat for longer, potentially reducing hunting pressure by reducing waste) (21, 101). Campaigns focused solely on minimizing risks to global public health ignore opportunities to improve healthcare for those at the forefront of the human-wildlife interface, often the same vulnerable communities most directly impacted by other conservation legislation (102).

Health rhetoric provides an alternative argument to reduce a person's impact on wildlife by promoting “Protect yourself” instead of “Protect the wildlife,” perhaps based on the assumption that the latter is not intrinsically important to the individual. However, there is a debate about whether we need health messaging to achieve conservation outcomes. Following the World Wildlife Fund Zero Wild Meat campaign, preserving nature remained the main reason people in Vietnam and the Lao People's Democratic Republic intended to abstain from eating wild meat (103). In a study of local risk perceptions associated with wild meat in Tanzania-Kenya (80), 62% of 299 people agreed that wild meat should not be sold because of disease risk, representing less than the 69% who believed it should not be sold for conservation reasons. Regardless of motivations, the majority (81%) stated they would stop buying wild meat if there were a cheaper alternative. Identifying barriers that prevent people from reducing their wild meat consumption (e.g. price, access, quality, values) can help inform conservation measures without relying on inappropriate public health messaging. This is not to say that conservation organizations cannot play a role in reducing the risk of zoonotic spillover for human health. Biodiversity declines, land-use change, urbanization, changes in connectivity, wildlife trade, and climate change have all been shown to impact disease dynamics and increase the likelihood of future spillover events (52, 104–106). Therefore, conservation actions that promote ecosystem health and reduce the rate of global warming help safeguard human health more broadly and help conserve wildlife populations. In addition, conservation has an important role in preventing **reverse spillover events** and spillover from captive to wild animals which pose significant threats to wildlife populations (23 , 107). Thus, lessons and practices from conservation are vital for policymakers seeking to balance human, animal, and ecosystem health. However, leveraging public health fears to fund and justify strict conservation practices may ultimately undermine core conservation values, reducing societal tolerance of wildlife, and amplifying existing conflicts between authorities and local communities (61, 102, 108). In places where risks of undernutrition are high and wildlife contributes substantially to nutrient intake, restricting access to wildlife can undermine current health in the name of reducing potential risks in the future. Therefore, bans on wild meat consumption can have counterproductive consequences for conservation and human health (26, 108–110).

### Implications for risk management and the need for evidence-based strategies

#### Improving scientific reporting

The complexities of understanding zoonotic disease transmission in socio-ecological systems have hindered our ability to effectively assess, communicate, and manage health risks linked to wild meat. We call for clearer definitions of zoonotic terms across studies to enable better comparison. Simply defining a pathogen as zoonotic is insufficient to gauge its human health impact, as spillover risk depends on many factors including exposure likelihood. Rather than creating more complex terminology to differentiate types of zoonoses [e.g. Singh et al. (25 )], we advocate for a tiered approach that considers transmission likelihood and health impacts [e.g. Grange et al. (111)]. Ideally, risk assessments would consider multiple information criteria to determine the relative health risks linked to different stages in the wild meat supply chain, including infectious agent taxonomy, an agent's capacity to infect humans, subsequent health implications, known transmission pathways, the proportion of infections associated with zoonotic transmission versus human-to-human transmission, and relevant host information such as agent prevalence and host ecology. Including this detail of information will not be possible in all circumstances but would serve as a useful framework to identify knowledge gaps. In all cases, studies should at least specify whether they are focused on agents, infections, or diseases; define their terms; and state the quality of evidence included in the data. Studies must acknowledge the uncertainties associated with the criteria above. Without such information, study findings may be incorrectly interpreted.

#### Contextualizing wild meat within food systems

Domestic animals can and do act as important reservoirs, intermediary hosts, or amplifier hosts of zoonotic pathogens (53). Livestock production occurs at industrial scales, raising animals in high concentrations with a focus on maximizing profits often at the expense of biosecurity, environmental sustainability, and animal welfare. Livestock production also provides greater opportunities for exposure given close, frequent contact between livestock and humans during animal rearing. Therefore, disease risks of consuming wild meat should be considered within the broader context of food safety and animal husbandry to prevent misrepresentation. Plourde et al. (112), Gibb et al. (52), Carlson et al. (113), GIDEON (41), and Zhou et al. (114) all provide data on infectious agents shared between animals and humans. However, not accounting for additional disease dynamics can inflate zoonotic disease risk associated with wildlife. When studies aim to inform the management of wild meat consumption, local clinical and structural vulnerabilities in the supply chain should be considered. Social, economic, and environmental factors impact people's ability to interpret risk and their knowledge of how to protect themselves, as well as having the resources to do so (115). Wild meat markets are not homogeneous, nor are motivations behind wild meat consumption (7, 24, 56). Therefore, different contexts require different interventions to promote safe and sustainable food consumption.

#### Ensuring transparency and addressing bias

Transparency also means communicating an organization's or researcher's positionality, especially as we seek to increase biosurveillance capacities (102, 116). Recognizing and respecting local food sovereignty should be at the heart of food policies, including for wild meat (117). However, policymakers must balance sociocultural and health needs with environmental sustainability and disease risk, given the different roles that wild meat plays in people's diets (i.e. from subsistence to luxury goods) (104). Arguments for banning hunting and wild meat consumption can stem from **animal welfare** concerns, rather than species conservation. Because transport/market conditions affect the risk of zoonotic transmission, establishing animal welfare standards for the legal and sustainable sale of wild meat is important to reduce disease spread. However, welfare arguments focus on the ethical implications of handling, housing, and killing animals for food. While animal welfare presents a legitimate rationale against wild meat consumption, animal welfare policies should not be disguised as those protecting species conservation or human health (88).

Public trust is critical in campaigns for behavior change for public health or conservation (118, 119). Public trust can be quickly eroded when a messenger is perceived to have a competing political, social, or economic agenda (97). The conservation sector must acknowledge how a legacy of current and historical injustices against local and Indigenous communities, and more broadly those in the Global South, has impacted public trust (89, 119). Therefore, strategies implemented to reduce health risks must be based on robust evidence and, wherever possible, be co-designed by those they ultimately affect. Co-design should be open to mutual knowledge exchange, learning about existing practices used by communities to safeguard health. Co-design demonstrates a meaningful effort to meet the needs and values of the people involved, re-establishing trust and communication, while promoting safe, sustainable use (24, 61, 95, 120). During acute public health crises, rapid policy decisions may be necessary without consultation. In these circumstances, transparency relies on honest, clear communication of how decisions are being made and what we do and do not know (24, 118, 121).

## Conclusion

Appropriate and well-informed management of wild meat is not only imperative for managing the risks of infectious disease to human health, locally and internationally, but also to ensure food security, public relations, and positive conservation outcomes. Improving our understanding of zoonotic disease risks will require more consistent terminology and clear, transparent communication to ensure effective solutions that balance environmental, human, and animal health in different socioecological contexts. When determining risk, studies must clarify how risk is defined and acknowledge that increased agent diversity does not necessarily equate to human health impacts. Wild meat is just one component of much larger food systems, and wild meat-health research needs to be better contextualized within these systems.

## Data Availability

There are no data underlying this work.

## References

[1] van Seventer JM, Hochberg NS. 2017. Principles of infectious diseases: transmission, diagnosis, prevention, and control. Int Encycl Public Health. 22–39. 10.1016/B978-0-12-803678-5.00516-6.

[2] Haydon DT, Cleaveland S, Taylor LH, Laurenson MK. 2002. Identifying reservoirs of infection: a conceptual and practical challenge. Emerg Infect Dis. 8(12):1468–1473. 10.3201/eid0812.010317. 12498665 PMC2738515

[3] Salkeld D, Hopkins S, Hayman D, editors. Identifying animal reservoirs during an epidemic. In: Emerging zoonotic and wildlife pathogens: disease ecology, epidemiology, and conservation. Oxford University Press, New York, USA, 2023. p. 155–172.

[4] Quesada J, Hart LK, Bourgois P. 2011. Structural vulnerability and health: Latino migrant laborers in the United States. Med Anthropol. 30(4):339–362. 10.1016/10.1080/01459740.2011.576725. 21777121 PMC3146033

[5] Wilson AJ, et al 2017. What is a vector? Philos Trans R Soc Lond B Biol Sci. 372(1719):20160085. 10.1016/10.1098/rstb.2016.0085. 28289253 PMC5352812

[6] World Health Organization . Revised draft of the negotiating text of the WHO pandemic agreement. WHO Press, Geneva, Switzerland, 2024.

[7] Lin B, Dietrich ML, Senior RA, Wilcove DS. 2021. A better classification of wet markets is key to safeguarding human health and biodiversity. Lancet Planet Health. 5:e386–e394. 34119013 10.1016/S2542-5196(21)00112-1 PMC8578676

[8] Ingram DJ, et al 2021. Wild meat is still on the menu: progress in wild meat research, policy, and practice from 2002 to 2020. Annu Rev Environ Resour. 46:221–254.

[9] Jones KE, et al 2008. Global trends in emerging infectious diseases. Nature. 451:990–993. 18288193 10.1038/nature06536 PMC5960580

[10] Woolhouse MEJ, Gowtage-Sequeria S. 2005. Host range and emerging and reemerging pathogens. Emerg Infect Dis. 11:1842–1847. 16485468 10.3201/eid1112.050997 PMC3367654

[11] Taylor LH, Latham SM, Woolhouse MEJ. 2001. Risk factors for human disease emergence. Phil Trans R Soc Lond B. 356:983–989. 11516376 10.1098/rstb.2001.0888 PMC1088493

[12] Dattani S, Rodés-Guirao L, Mathieu E, Ritchie H, Roser M. Pandemics. 2023. [accessed •••]. https://ourworldindata.org/pandemics.

[13] Reed KD . Viral zoonoses. In: Reference module in biomedical sciences. Elsevier, 2018. doi:10.1016/B978-0-12-801238-3.95729-5

[14] The Lancet Public Health . 2020. COVID-19 puts societies to the test. Lancet Public Health. 5:e235. 32380034 10.1016/S2468-2667(20)30097-9 PMC7198208

[15] Verschuur J, Koks EE, Hall JW. 2021. Observed impacts of the COVID-19 pandemic on global trade. Nat Hum Behav. 5:305–307. 33633376 10.1038/s41562-021-01060-5

[16] Bonwitt J, et al 2018. Unintended consequences of the ‘bushmeat ban’ in West Africa during the 2013–2016 Ebola virus disease epidemic. Soc Sci Med. 200:166–173. 29421463 10.1016/j.socscimed.2017.12.028

[17] Roe D, et al 2020. Beyond banning wildlife trade: COVID-19, conservation and development. World Dev. 136:105121. 32834392 10.1016/j.worlddev.2020.105121 PMC7388857

[18] Koh LP, Li Y, Lee JSH. 2021. The value of China's ban on wildlife trade and consumption. Nat Sustain. 4:2–4.

[19] AFP . 2020. Gabon bans eating of pangolin and bats amid pandemic. https://phys.org/news/2020-04-gabon-pangolin-pandemic.html. Accessed 24 January 2024.

[20] Golden CD, Fernald LCH, Brashares JS, Rasolofoniaina BJR, Kremen C. 2011. Benefits of wildlife consumption to child nutrition in a biodiversity hotspot. Proc Natl Acad Sci U S A. 108:19653–19656. 22106297 10.1073/pnas.1112586108 PMC3241784

[21] Van Vliet N, et al 2017. Bushmeat and human health: assessing the evidence in tropical and sub-tropical forests. Ethnobiol Conserv. 6:3.

[22] Morsello C, et al 2015. Cultural attitudes are stronger predictors of bushmeat consumption and preference than economic factors among urban Amazonians from Brazil and Colombia. Ecol Soc. 20:21.

[23] IUCN, EcoHealth Alliance . PANORAMA solutions in focus: wildlife health and zoonotic disease risk reduction. IUCN and EcoHealth Alliance, 2022.

[24] World Organisation for Animal Health. Guidelines for addressing disease risks in wildlife trade. World Organisation for Animal Health, 2024.

[25] Singh BB, Ward MP, Kostoulas P, Dhand NK. 2023. Zoonosis–why we should reconsider “What's in a name?” Front Public Health. 11:1133330. 36860402 10.3389/fpubh.2023.1133330 PMC9969093

[26] Eskew EA, Carlson CJ. 2020. Overselling wildlife trade bans will not bolster conservation or pandemic preparedness. Lancet Planet Health. 4:e215–e216. 32497492 10.1016/S2542-5196(20)30123-6 PMC7263806

[27] World Health Organisation . World malaria report. World Health Organisation, Geneva, Switzerland, 2023.

[28] Moyes CL, et al 2014. Defining the geographical range of the Plasmodium knowlesi reservoir. PLoS Negl Trop Dis. 8(3):e2780. 10.1016/10.1371/journal.pntd.0002780. 24676231 PMC3967999

[29] Ta TH, et al 2014. First case of a naturally acquired human infection with Plasmodium cynomolgi. Malar J. 13(1):68. 10.1016/10.1186/1475-2875-13-68. 24564912 PMC3937822

[30] Lalremruata A, et al 2015. Natural infection of Plasmodium brasilianum in humans: man and monkey share quartan malaria parasites in the Venezuelan Amazon. EBioMedicine. 2(9):1186–1192. 10.1016/10.1016/j.ebiom.2015.07.033. 26501116 PMC4588399

[31] Brasil P, et al 2017. Outbreak of human malaria caused by Plasmodium simium in the Atlantic Forest in Rio de Janeiro: a molecular epidemiological investigation. Lancet Glob Health. 5(10):e1038–e1046. 10.1016/10.1016/S2214-109X(17)30333-9. 28867401

[32] Prugnolle F, et al 2013. Diversity, host switching and evolution of Plasmodium vivax infecting African great apes. Proc Natl Acad Sci U S A. 110(20):8123–8128. 10.1016/10.1073/pnas.1306004110. 23637341 PMC3657773

[33] Fornace KM, Drakeley CJ, Lindblade KA, Jelip J, Ahmed K. 2023. Zoonotic malaria requires new policy approaches to malaria elimination. Nat Commun. 14(1):5750. 10.1016/10.1038/s41467-023-41546-6. 37717079 PMC10505154

[34] Kuiken T, et al 2003. Newly discovered coronavirus as the primary cause of severe acute respiratory syndrome. Lancet. 362:263–270. 12892955 10.1016/S0140-6736(03)13967-0 PMC7112434

[35] World Health Organization. WHO-convened global study of origins of SARS-CoV-2: China part. WHO Press, 2021.

[36] PREDICT Project . 2019. *UC Davis One Health Institute School of Veterinary Medicine*. https://ohi.vetmed.ucdavis.edu/programs-projects/predict-project. Accessed 30 July 2024.

[37] World Organisation for Animal Health . Chapter 2.2.7. Principles and methods for the validation of diagnostic tests for infectious diseases applicable to wildlife. In: OIE Manual of diagnostic tests and vaccines for terrestrial animals. 8th Ed. World Organisation for Animal Health, Paris, France, 2018. p. 1–12.

[38] Mollentze N, Babayan SA, Streicker DG. 2021. Identifying and prioritizing potential human-infecting viruses from their genome sequences. PLoS Biol. 19(9):e3001390. 10.1016/10.1371/journal.pbio.3001390 34582436 PMC8478193

[39] Mollentze N, Streicker DG. 2023. Predicting zoonotic potential of viruses: where are we? Curr Opin Virol. 61:101346. 37515983 10.1016/j.coviro.2023.101346

[40] Shaw LP, et al 2020. The phylogenetic range of bacterial and viral pathogens of vertebrates. Mol Ecol. 29:3361–3379. 32390272 10.1111/mec.15463

[41] GIDEON . 2024. GIDEON Infectious Disease Database. https://app.gideononline.com/login. Accessed 6 March 2024.

[42] Bansal N, Raturi M, Bansal Y. 2022. SARS-CoV-2 variants in immunocompromised COVID-19 patients: the underlying causes and the way forward. Transfus Clin Biol. 29:161–163. 34973463 10.1016/j.tracli.2021.12.006 PMC8714679

[43] Milstein MS, et al 2020. An ethnographic approach to characterizing potential pathways of zoonotic disease transmission from wild meat in Guyana. EcoHealth. 17:424–436. 33792854 10.1007/s10393-021-01513-3

[44] Jenkins J, Lawundeh W, Hanson T, Brown H. 2024. Human-animal entanglements in bushmeat trading in Sierra Leone: an ethnographic assessment of a potential zoonotic interface. PLoS One. 19:e0298929. 38547141 10.1371/journal.pone.0298929 PMC10977710

[45] Tumelty L, et al 2023. A systematic mapping review of links between handling wild meat and zoonotic diseases. One Health. 17:100637. 10.1016/j.onehlt.2023.100637 38024256 PMC10665173

[46] Peros CS, Dasgupta R, Kumar P, Johnson BA. 2021. Bushmeat, wet markets, and the risks of pandemics: exploring the nexus through systematic review of scientific disclosures. Environ Sci Policy. 124:1–11. 36536884 10.1016/j.envsci.2021.05.025 PMC9751798

[47] Milbank C, Vira B. 2022. Wildmeat consumption and zoonotic spillover: contextualising disease emergence and policy responses. Lancet Planet Health. 6:e439–e448. 35550083 10.1016/S2542-5196(22)00064-X PMC9084621

[48] Moloney GK, Gaubert P, Gryseels S, Verheyen E, Chaber A-L. 2023. Investigating infectious organisms of public health concern associated with wild meat. Transbound Emerg Dis. 2023:e5901974. 10.1155/2023/5901974 PMC12017266 40303741

[49] Choo J, Nghiem LTP, Chng S, Carrasco LR, Benítez-López A. 2024. Hotspots of zoonotic disease risk from wildlife hunting and trade in the tropics. Integr Conserv. 2:165–175.

[50] Jagadesh S, Zhao C, Mulchandani R, Boeckel TPV. 2023. Mapping global bushmeat activities to improve zoonotic spillover surveillance by using geospatial modeling. Emerg Infect Dis. 29:742–750. 36957996 10.3201/eid2904.221022 PMC10045693

[51] Allen T, et al 2017. Global hotspots and correlates of emerging zoonotic diseases. Nat Commun. 8:1124. 29066781 10.1038/s41467-017-00923-8 PMC5654761

[52] Gibb R, et al 2020. Zoonotic host diversity increases in human-dominated ecosystems. Nature. 584:398–402. 32759999 10.1038/s41586-020-2562-8

[53] Otte J, Pica-Ciamarra U. 2021. Emerging infectious zoonotic diseases: the neglected role of food animals. One Health. 13:100323. 34522761 10.1016/j.onehlt.2021.100323 PMC8426280

[54] Rostagno MH . 2009. Can stress in farm animals increase food safety risk? Foodborne Pathog Dis. 6:767–776. 19737056 10.1089/fpd.2009.0315

[55] The Lancet . 2024. H5n1: international failures and uncomfortable truths. Lancet. 403:2455. 38851277 10.1016/S0140-6736(24)01184-X

[56] Campbell S, et al Options for managing and tracing wild animal trade chains to reduce zoonotic risk 2022. [accessed 2024 Jun 27]. https://www.traffic.org/publications/reports/review-options-for-managing-and-tracing-wild-animal-trade-chains-to-reduce-zoonotic-risk/.

[57] Liebman AK, Juarez-Carrillo PM, Reyes IAC, Keifer MC. 2016. Immigrant dairy workers’ perceptions of health and safety on the farm in America's Heartland. Am J Ind Med. 59:227–235. 26523613 10.1002/ajim.22538

[58] Bloss JE, et al 2022. Advancing the health of migrant and seasonal farmworkers in the United States: identifying gaps in the existing literature, 2021. Health Promot Pract. 23:432–444. 34549654 10.1177/15248399211033308 PMC9096586

[59] World Health Organization . WHO Estimates of the global burden of foodborne diseases: foodborne disease burden epidemiology reference group 2007–2015. WHO Press, 2015.

[60] Karesh WB, et al 2012. Ecology of zoonoses: natural and unnatural histories. Lancet. 380:1936–1945. 23200502 10.1016/S0140-6736(12)61678-X PMC7138068

[61] Zhou W, Orrick K, Lim A, Dove M. 2021. Reframing conservation and development perspectives on bushmeat. Environ Res Lett. 17:011001.

[62] Peacock TP, et al 2025. The global H5N1 influenza panzootic in mammals. Nature. 637:304–313. 39317240 10.1038/s41586-024-08054-z

[63] Nguyen T-Q, et al 2025. Emergence and interstate spread of highly pathogenic avian influenza A (H5N1) in dairy cattle in the United States. Science. 388:eadq0900. 40273240 10.1126/science.adq0900

[64] Lipkin WI . Zoonoses. In: Bennett JE, Dolin R, Blaser MJ, editors. Mandell, Douglas, and Bennett's Principles and practice of infectious diseases. Elsevier; 2015. p. 3554–3558.

[65] Halonen SK, Weiss LM. 2013. Toxoplasmosis. Handb Clin Neurol. 114:125–145. 23829904 10.1016/B978-0-444-53490-3.00008-X PMC4157368

[66] Torgerson PR, Macpherson CNL. 2011. The socioeconomic burden of parasitic zoonoses: global trends. Vet Parasitol. 182:79–95. 21862222 10.1016/j.vetpar.2011.07.017

[67] World Health Organization. Communicable diseases and severe food shortage: WHO technical note. WHO Press, 2010. 26158188

[68] Sinha P, Guerrant RL. 2023. The costly vicious cycle of infections and malnutrition. J Infect Dis. 229:1611–1613. 10.1093/infdis/jiad513 PMC11175688 37972258

[69] Blaney S, Beaudry M, Latham M. 2009. Contribution of natural resources to nutritional status in a protected area of Gabon. Food Nutr Bull. 30:49–62. 19445259 10.1177/156482650903000105

[70] Carignano Torres P, et al 2022. Wildmeat consumption and child health in Amazonia. Sci Rep. 12:5213. 35388037 10.1038/s41598-022-09260-3 PMC8986765

[71] Duonamou L, et al 2020. Consumer perceptions and reported wild and domestic meat and fish consumption behavior during the Ebola epidemic in Guinea, West Africa. PeerJ. 8:e9229. 32566394 10.7717/peerj.9229 PMC7293194

[72] Niewiadomska K, Kosicka-Gębska M, Gębski J, Jeżewska-Zychowicz M, Sułek M. 2021. Perception of the health threats related to the consumption of wild animal meat—is eating game risky? Foods. 10:1544. 34359415 10.3390/foods10071544 PMC8303633

[73] Tomasevic I, et al 2018. Consumers’ perceptions, attitudes and perceived quality of game meat in ten European countries. Meat Sci. 142:5–13. 29635220 10.1016/j.meatsci.2018.03.016

[74] Chausson AM, Rowcliffe JM, Escouflaire L, Wieland M, Wright JH. 2019. Understanding the sociocultural drivers of urban bushmeat consumption for behavior change interventions in Pointe Noire, Republic of Congo. Hum Ecol. 47:179–191.

[75] Xie X, Huang L, Li JJ, Zhu H. 2020. Generational differences in perceptions of food health/risk and attitudes toward organic food and game meat: the case of the COVID-19 crisis in China. Int J Environ Res Public Health. 17:3148. 32366016 10.3390/ijerph17093148 PMC7246561

[76] Brittain S, et al 2022. The drivers of wild meat consumption in rural Cameroon: insights for wild meat alternative project design. Conserv Sci Pract. 4:e12700.

[77] Blum L, Khan R, Nahar N, Breiman R. 2009. In-depth assessment of an outbreak of nipah encephalitis with person-to-person transmission in Bangladesh: implications for prevention and control strategies. Am J Trop Med Hyg. 80:96–102. 19141846

[78] Akem ES, Pemunta NV. 2020. The bat meat chain and perceptions of the risk of contracting Ebola in the Mount Cameroon region. BMC Public Health. 20:593. 32354371 10.1186/s12889-020-08460-8 PMC7193336

[79] Vaske JJ, Miller CA. 2018. Hunters and non-hunters chronic wasting disease risk perceptions over time. Soc Nat Resour. 31:1379–1388.

[80] Patel EH, et al 2023. Assessing disease risk perceptions of wild meat in savanna borderland settlements in Kenya and Tanzania. Front Ecol Evol. 11:1033336.

[81] Worobey M, et al 2022. The huanan seafood wholesale market in Wuhan was the early epicenter of the COVID-19 pandemic. Science. 377:951–959. 35881010 10.1126/science.abp8715 PMC9348750

[82] Xiao X, Newman C, Buesching CD, Macdonald DW, Zhou Z-M. 2021. Animal sales from Wuhan wet markets immediately prior to the COVID-19 pandemic. Sci Rep. 11:11898. 34099828 10.1038/s41598-021-91470-2 PMC8184983

[83] Walzer C, Kang A. 2020. Abolish Asia's ‘wet markets,’ where pandemics breed. Wall Street Journal. [accessed 2024 Feb 14]. https://www.wsj.com/articles/abolish-asias-wet-markets-where-pandemics-breed-11580168707.

[84] Forgey Q . 2020. Shut down those things right away’: calls to close ‘wet markets’ ramp up pressure on China. POLITICO. [accessed 2024 Feb 14]. https://www.politico.com/news/2020/04/03/anthony-fauci-foreign-wet-markets-shutdown-162975.

[85] Greenfield P . 2020. Ban wildlife markets to avert pandemics, says UN biodiversity chief. Guardian. [accessed 2024 Feb 14]. https://www.theguardian.com/world/2020/apr/06/ban-live-animal-markets-pandemics-un-biodiversity-chief-age-of-extinction.

[86] Crits-Christoph A, et al 2024. Genetic tracing of market wildlife and viruses at the epicenter of the COVID-19 pandemic. Cell. 187:5468–5482.e11. 39303692 10.1016/j.cell.2024.08.010 PMC11427129

[87] Liu WJ, et al 2024. Surveillance of SARS-CoV-2 at the huanan seafood market. Nature. 631:402–408. 37019149 10.1038/s41586-023-06043-2

[88] Muller SM . 2021. Carnistic colonialism: a rhetorical dissection of “bushmeat” in the 2014 Ebola outbreak. Front Commun. 6:656431.

[89] Rudd LF, et al 2021. Overcoming racism in the twin spheres of conservation science and practice. Proc R Soc Lond B Biol Sci. 288:20211871. 10.1098/rspb.2021.1871 PMC8564623 34727721

[90] León B, López-Goñi I, Salaverría R. 2022. The COVID-19 catastrophe: a science communication mess? Church Commun Culture. 7:6–22.

[91] Komesaroff PA, Dwyer DE. 2023. The question of the origins of COVID-19 and the ends of science. J Bioeth Inq. 20:575–583. 10.1007/s11673-023-10303-1 37697176 PMC10942872

[92] Benítez-López A, et al 2017. The impact of hunting on tropical mammal and bird populations. Science. 356:180–183. 28408600 10.1126/science.aaj1891

[93] Rizvi DS . 2022. Health education and global health: practices, applications, and future research. J Educ Health Promot. 11:262. 36325224 10.4103/jehp.jehp_218_22 PMC9621358

[94] Arlinghaus KR, Johnston CA. 2017. Advocating for behavior change with education. Am J Lifestyle Med. 12:113–116. 30283247 10.1177/1559827617745479 PMC6124997

[95] Thung PH, Chua L. 2020. Why COVID-era campaigns against wildmeat consumption aren’t working. The Conversation. [accessed 2024 Feb 14]. https://theconversation.com/why-covid-era-campaigns-against-wildmeat-consumption-arent-working-148474.

[96] Saylors KE, et al 2021. Market characteristics and zoonotic disease risk perception in Cameroon bushmeat markets. Soc Sci Med. 268:113358. 32992090 10.1016/j.socscimed.2020.113358

[97] Leiss W . 2006. Down and dirty: the use and abuse of public trust in risk communication. Risk Anal. 15:685–692.

[98] Friant S, et al 2020. Eating bushmeat improves food security in a biodiversity and infectious disease “hotspot.” EcoHealth. 17:125–138. 32020354 10.1007/s10393-020-01473-0

[99] Petrovan SO, et al 2021. Post COVID-19: a solution scan of options for preventing future zoonotic epidemics. Biol Rev Camb Philos Soc. 96:2694–2715. 34231315 10.1111/brv.12774 PMC8444924

[100] Hilderink MH, de Winter II. 2021. No need to beat around the bushmeat–the role of wildlife trade and conservation initiatives in the emergence of zoonotic diseases. Heliyon. 7:e07692. 34386637 10.1016/j.heliyon.2021.e07692 PMC8342965

[101] Buck AJ, Tchai T, Spiegel U, Morra WA. 2017. Refrigeration and the reduction of the takeoff rate of bushmeat. Sage Open. 7:2158244016684174.

[102] Smith W . 2022. Understanding the changing role of global public health in biodiversity conservation. Ambio. 51:485–493. 34115346 10.1007/s13280-021-01576-0 PMC8194382

[103] World Wildlife Fund D . Zero wild meat campaign: through a behavior change lens. World Wildlife Fund, Switzerland, 2023. https://www.worldwildlife.org/publications/zero-wild-meat-campaign-through-a-behavior-change-lens/.

[104] Ingram DJ . 2020. Wild meat in changing times. J Ethnobiol. 40:117–130.

[105] Gibb R, et al 22 May 2024. The anthropogenic fingerprint on emerging infectious diseases. medRxiv. 10.1101/2024.05.22.24307684, preprint: not peer reviewed.

[106] Redding DW, Gibb R, Jones KE. 11 February 2024. Ecological impacts of climate change will transform public health priorities for zoonotic and vector-borne disease. medRxiv 24302575. 10.1101/2024.02.09.24302575, preprint: not peer reviewed.

[107] Fagre AC, et al 2022. Assessing the risk of human-to-wildlife pathogen transmission for conservation and public health. Ecol Lett. 25:1534–1549. 35318793 10.1111/ele.14003 PMC9313783

[108] Buttke DE, Decker DJ, Wild MA. 2015. The role of one health in wildlife conservation: a challenge and opportunity. J Wildl Dis. 51:1–8. 25375941 10.7589/2014-01-004

[109] Cronin DT, et al 2015. Long-term urban market dynamics reveal increased bushmeat carcass volume despite economic growth and proactive environmental legislation on Bioko Island, Equatorial Guinea. PLoS One. 10:e0134464. 26230504 10.1371/journal.pone.0134464 PMC4521855

[110] Roe D, Lee TM. 2021. Possible negative consequences of a wildlife trade ban. Nat Sustain. 4:5–6.

[111] Grange ZL, et al 2021. Ranking the risk of animal-to-human spillover for newly discovered viruses. Proc Natl Acad Sci U S A. 118:e2002324118. 33822740 10.1073/pnas.2002324118 PMC8053939

[112] Plourde BT, et al 2017. Are disease reservoirs special? Taxonomic and life history characteristics. PLoS One. 12:e0180716. 28704402 10.1371/journal.pone.0180716 PMC5509157

[113] Carlson CJ, et al 2022. The global virome in one network (VIRION): an atlas of vertebrate-virus associations. mBio. 13:e02985-21. 35229639 10.1128/mbio.02985-21 PMC8941870

[114] Zhou S, et al 2022. ZOVER: the database of zoonotic and vector-borne viruses. Nucleic Acids Res. 50:D943–D949. 34634795 10.1093/nar/gkab862 PMC8728136

[115] Robert E, et al 2021. Environmental determinants of E. coli, link with the diarrheal diseases, and indication of vulnerability criteria in tropical West Africa (Kapore, Burkina Faso). PLoS Negl Trop Dis. 15:e0009634. 34403418 10.1371/journal.pntd.0009634 PMC8370611

[116] Gregg EA, et al 2022. Ethical considerations for conservation messaging research and practice. People Nat. 4:1098–1112.

[117] Van Vliet N . 2018. “Bushmeat crisis” and “cultural imperialism” in wildlife management? Taking value orientations into account for a more sustainable and culturally acceptable wildmeat sector. Front Ecol Evol. 6:112.

[118] Hyland-Wood B, Gardner J, Leask J, Ecker UKH. 2021. Toward effective government communication strategies in the era of COVID-19. Humanit Soc Sci Commun. 8:30.

[119] Saif O, Keane A, Staddon S. 2022. Making a case for the consideration of trust, justice, and power in conservation relationships. Conserv Biol. 36:e13903. 35212065 10.1111/cobi.13903 PMC9545749

[120] WCS Rights and Communities Team . The COVID-19 pandemic and indigenous peoples and local communities: protecting people, protecting rights. Wildlife Conservation Society, New York, USA, 2020. https://cdn.wcs.org/2020/06/29/66o8ub6lop_WCS_COVID_19_PandemicAndIndigenousPeoples.pdf.

[121] Shapiro JT, et al 2021. Setting the terms for zoonotic diseases: effective communication for research, conservation, and public policy. Viruses. 13:1356. 34372562 10.3390/v13071356 PMC8310020

